# A Foreigner's View on English Cottage Hospitals

**Published:** 1912-02-03

**Authors:** Mario Ballarelli

**Affiliations:** Rag. Cardiff


### The Editor's Letter Box.

### A FOREIGNER'S VIEW ON ENGLISH COTTAGE HOSPITALS.

*To the Editor of* The Hospital.

Sir,—Will you accept the best expressions of my sympathy for your critique contained in the last number of The Hospital against a recent book on "The Cottage Hospital."

I am a foreigner, reader of your newspaper, and I take the greatest interest to the hospital organisation in England. I paid visits to many hospitals in this country, both in towns and in small centers. Everywhere I had reason to feel quite amazed for the wonderful organisation of these charitable institutions, and my studying has strengthened my opinion, especially, I am glad to say, for the hospital of the small centers.

You will therefore understand how hurted I felt when I had the misfortune of reading the book above referred to, and how grateful I am now to you for your severe judgement of it.

It is really a pity that this book has been written with so many precautions as to prevent any action for slander.

If you think that an uninterested opinion of a foreigner, admirer of all the forms of British charity will be of any use against the pernicious action of such a libel, I authorise you to publish these few lines on your paper.

Believe me, dear Sir, yours truly,

Mario Ballarelli, Rag.

Cardiff, January 29.

